# Relationship between serum uric acid and hypertension: a cross-sectional study in Bangladeshi adults

**DOI:** 10.1038/s41598-019-45680-4

**Published:** 2019-06-21

**Authors:** Nurshad Ali, Shakil Mahmood, Farjana Islam, Shahnaz Rahman, Tangigul Haque, Shiful Islam, Sadaqur Rahman, Nazmul Haque, Golam Mosaib, Rasheda Perveen, Farida Adib Khanum

**Affiliations:** 1 Department of Biochemistry and Molecular Biology, Shahjalal University of Science and Technology, Sylhet, 3114 Bangladesh; 2 Department of Biochemistry and Molecular Biology, Gono University and Gonoshasthaya Samaj Vittik Medical College, Savar, Dhaka 1344 Bangladesh

**Keywords:** Disease prevention, Interventional cardiology

## Abstract

Experimental evidence suggests a causal role of serum uric acid (SUA) in hypertension development. Currently, there are few data available on the association between SUA and hypertension; data from Bangladeshi adults are not available yet. This study evaluated the association of SUA with hypertension among Bangladeshi adults. Blood samples were obtained from 140 males and 115 females and analyzed for SUA and lipid levels. Hypertension was defined as SBP ≥ 140 mmHg and/or DBP ≥ 90 mmHg. All participants were divided into four quartiles based on SUA concentrations. Association of SUA with hypertension was evaluated by logistic regression models. The prevalence of hypertension and prehypertension was significantly higher in male (15.4 and 47.6%, respectively) than in the female (5.6 and 33.4%, respectively) subjects (p < 0.01). Males had a higher mean level of SUA (310.7 ± 67.9 µmol/L) than in the females (255.3 ± 69.3 µmol/L) (p < 0.001). Hyperuricemia was prevalent 9.1% in males and 10.3% in females. An increasing trend for hypertension and prehypertension was found in both genders with increasing SUA levels in the quartiles (p < 0.01). SUA levels in the quartiles were positively correlated with blood pressure (p < 0.01). After adjusting for baseline covariates, SUA levels were significantly associated with hypertension (p < 0.01). Findings of this study indicate the significance of maintaining normal SUA level to prevent hypertension.

## Introduction

The prevalence of hypertension is increasing worldwide and is an important contributor to cardiovascular diseases (CVDs) and premature death^1,2^. In the past years, an upward trend of hypertension prevalence has been reported in general and young adults in Bangladesh^3–5^.

Serum uric acid (SUA) is the end product of purine metabolism; it’s over production and decreased excretion via kidneys are one of the main causes of hyperuricemia in human^6^. So far, there are three uric acid/urate transporters (URAT1/SLC22A12, GLUT9/SLC2A9, and ABCG2/BCRP) that have been reported to play crucial roles in the regulation of SUA and their abnormal functions lead to hypouricemia and/or hyperuricemia^7–11^. Among them, common abnormalities of ABCG2 exporter has been shown to be a major cause of hyperuricemia and gout^7,11^. It has been reported that ABCG2 dysfunction causes renal urate underexcretion and inducing hyperuricemia^12^. In addition, ABCG2 variants have been reported to show stronger effects on hyperuricemia than main environmental risk factors such as obesity, age, and alcohol drinking^13^.

Hyperuricemia prevalence is increasing rapidly in the world communities; emerging evidence indicates that hyperuricemia is now more common in the developing nations along with the developed nations^14^. Increased SUA has been found to be associated with CVDs, gout, metabolic syndrome and renal dysfunction^15–18^.

The link between increased SUA and various components of metabolic syndrome has been investigated in previous studies^13,19–21^. SUA was found to be positively associated with the number of metabolic syndrome included hypertension^20^. In recent years, the relationship of SUA with the incidence of hypertension has received widespread attention. An association between increased SUA and hypertension has been reported in some epidemiological studies^22–27^. It still remains unclear whether SUA is a risk factor, a mediator or merely a marker for hypertension in humans^28^. A few small-scale clinical trials have shown that SUA-lowering agents like probenecid and allopurinol are able to reduce blood pressure in adolescents^29,30^, suggesting that SUA might be an independent risk factor for hypertension. There are few studies that explored the relationship between SUA levels and hypertension in the general healthy population. To our knowledge, no studies have been conducted to explore the association between SUA and hypertension in Bangladeshi adults. Therefore, in this study, we aimed to investigate whether elevated SUA level is associated with hypertension in general adults in Bangladesh.

## Materials and Methods

### Study design and study subjects

This study was conducted between September 2017 and February 2018. A total of 255 subjects (140 males and 115 females; age >18 years) were enrolled in this study who were academic and non-academic staffs and young adult students of Gono University and Gonoshasthaya Samaj Vittik Medical College in Dhaka city and Shahjalal University of Science and Technology in Sylhet city of Bangladesh. All study subjects were informed about the study aims and written informed consent was obtained from them prior to enroll in the study. Individuals with having a history of gout and cardiac or severe renal diseases and anti-hyperuricemic and anti-hypertensive drugs intake were excluded from the study. The Ethics Committee at the Gonoshasthaya Samaj Vittik Medical College approved the study. All the steps of the methods section were conducted in accordance with the institutional guidelines and regulations.

### General data collection

Anthropometric indices of body weight (BW), body height (BH), hip circumference (HC), waist circumference (WC), and lifestyle information were recorded in a questionnaire described elsewhere^3,31^. Briefly, BW was measured to the nearest 0.1 kg using a calibrated digital weighing machine (Beurer 700, Germany) and BH was recorded to the nearest 0.1 cm using a height measuring tape. Body mass index (BMI) was calculated as the weight in kg divided by height in meter square. WC was measured by placing the tape horizontally midway between the iliac crest on the mid-auxiliary line and the ribs lowest border. HC was measured at the largest circumference of the buttocks.

### Measurements of blood pressure variables

Blood pressure (BP) was measured in all subjects by trained professionals using a digital BP machine (Omron M10, Omron Corporation, Tokyo, Japan). Blood pressure was measured on the left arm in a sitting position after the participant rested for 10 minutes. The first blood pressure measurement was discarded to avoid possible effects of anxiety, and the average value of second and third measurements was count for systolic and diastolic blood pressure (SBP and DBP). The participants were requested to avoid smoking, coffee, and tea for 30 minutes before blood pressure measurements.

### Blood samples and laboratory measurements

The venous blood sample was collected from each participant in the morning between 9:00 am to 10:00 am. After blood clotting, it was centrifuged and serum was stored at −20 °C until analysis. The concentration of SUA, and serum lipids: total cholesterol (TC), triglycerides (TG), low-density lipoprotein cholesterol (LDL-C), and high-density lipoprotein cholesterol (HDL-C), were determined calorimetrically using commercially available diagnostic kits (Human Diagnostic, Germany). All biochemical tests described above were measured in a biochemistry analyzer (Humalyzer 3000, USA). All laboratory tests were performed by trained professionals. Each serum sample was analyzed duplicate and average concentration used during calculation.

### Diagnostic criteria

Hypertension was defined as SBP ≥ 140 mm Hg and/or DBP ≥ 90 mm Hg and prehypertension is when SBP 120–139 mm Hg; and/or DBP 80–89 mm Hg^32^. Hyperuricemia was defined as SUA levels >416.4 µmol/L (7.0 mg/dL) in men and >356.9 µmol/L (6.0 mg/dL) in women^33,34^.

The participants were divided into four quartiles based on SUA levels (Q1: 119–226 µmol/L; Q2: 227–279 µmol/L; Q3: 280–339 µmol/L and Q4: 340–506 µmol/L) and the prevalence of hypertension and prehypertension was estimated in each quartile.

### Statistical analysis

The data were analyzed using IBM SPSS version 23. In tables, numeric data are summarized as mean ± SD. The difference between the sex groups for baseline variables was done by independent sample t-test (two-tailed). Pearson’s correlation coefficient test was performed to assess the interrelationships between baseline variables and SUA concentrations. The differences for variables among the groups were determined by One-way ANOVA. The relationship between SUA and hypertension was evaluated by logistic regression modeling. In model 1, age and sex were adjusted and model 2 was adjusted for age, sex, and BMI. Model 3 was further adjusted for variables used in model 1 and model 2 and lipid levels. A p-value < 0.05 was considered to be statistically significant.

## Results

### Baseline characteristics of the participants

Baseline characteristics of the participants are presented in Table 1. Of the 255 subjects, 140 were male, and 115 were female subjects. Mean age of the participants was 32.7 ± 13.8 years (range 18–80 years), with a significant difference between the gender groups (p < 0.01). There was no difference in the mean level of BMI between the male-female groups. Mean level of WC and HC was significantly different between male and female (p < 0.05) subjects. The mean level of SBP and DBP were significantly higher in the male subjects (p < 0.001). In Pearson’s correlation coefficient test, SUA levels were significantly associated with SBP and DBP (p < 0.001). Males had a higher mean level of SUA than in the female subjects (p < 0.001). The average level of TG and HDL-C were also significantly different between the gender groups (p < 0.001). Overall, hyperuricemia prevalence was 9.7% with 10.3% in females and 9.1% in males.

**Table 1:** Baseline characteristics of the study participants by gender.

Characteristics	Total (n = 255)		Male (n = 140)		Female (n = 115)		p-value
	Mean ± SD	Max	Mean ± SD	Max	Mean ± SD	Max	
Age, year	32.7 ± 13.8	80	35.7 ± 16.0	80	29.5 ± 10.0	62	0.002
BH, cm	160.3 ± 7.9	177	165.3 ± 5.2	177	153.2 ± 5.1	165	0.000
BW, kg	66.3 ± 9.9	93	66.8 ± 8.6	88	62.8 ± 10.7	93	0.000
WC, cm	69.6 ± 33.5	117	77.9 ± 25.9	106	60.5 ± 38.4	117	0.000
HC, cm	94.2 ± 7.8	124	92.9 ± 5.5	107	95.9 ± 9.9	124	0.017
BMI, kg/m^2^	24.7 ± 4.5	37	24.1 ± 4.7	32	25.4 ± 4.3	37	0.065
SUA, µmol/L	284.2 ± 73.8	506	310.7 ± 67.9	506	255.3 ± 69.3	441	0.000
Hyperuricemia, %	9.7	—	9.1	—	10.3	—	—
SBP, mm Hg	116.0 ± 14.6	174	121.8 ± 14.3	174	109.7 ± 12.2	156	0.000
DBP, mm Hg	75.0 ± 8.7	99	77.2 ± 7.8	99	72.6 ± 9.1	98	0.000
PP, mm Hg	66.7 ± 31.7	111	73.0 ± 21.5	103	59.8 ± 38.9	111	0.004
TG, mg/dL	153.3 ± 90.7	673	175.1 ± 89.5	360	129.5 ± 86.4	673	0.000
TC, mg/dL	140.2 ± 47.4	257	136.8 ± 51.6	257	143.9 ± 42.3	253	0.308
HDL-C, mg/dL	41.7 ± 13.5	88	37.8 ± 11.3	86	45.8 ± 14.5	88	0.000
LDL-C, mg/dL	74.2 ± 40.3	210	70.6 ± 42.9	210	78.1 ± 37.1	189	0.205

P-values are obtained from independent sample t-test in comparison between the gender groups.

### Levels of baseline variables in the SUA quartiles

Baseline information of the subjects in each SUA quartile is presented in Table 2. For all participants, an increasing trend for mean level of SUA was found across the quartiles (p < 0.01 for trend). The mean value of SBP and DBP was found to be increased with elevated concentrations of SUA in the quartiles (p < 0.01 for trend). The values of BMI, TC, TG, HDL-C, and LDL-C were increased with increasing concentrations of SUA (p < 0.05 for trend).

**Table 2:** Baseline characteristics of the study participants according to SUA quartiles.

	Q1 (119–226 µmol/L)	Q2 (227–279 µmol/L)	Q3 (280–339 µmol/L)	Q4 (340–506 µmol/L)	p-values for trend
*N*	64	65	64	62	—
Sex, m/f	22/42	31/34	40/24	47/15	—
Age, year	32.5 ± 11.8	32.5 ± 14.9	31.0 ± 14.8	32.1 ± 13.0	0.937
BH, cm	155.6 ± 7.8	158.0 ± 7.7	162.5 ± 6.5	165.1 ± 6.4	0.000
BW, kg	60.2 ± 10.0	63.5 ± 8.9	69.5 ± 9.6	71.9 ± 7.3	0.000
WC, cm	66.2 ± 29.8	68.0 ± 36.3	68.7 ± 35.9	72.8 ± 33.5	0.048
HC, cm	91.3 ± 7.8	93.8 ± 8.6	95.5 ± 6.8	96.1 ± 7.4	0.037
BMI, kg/m^2^	24.1 ± 3.8	24.2 ± 3.4	24.7 ± 6.2	25.9 ± 3.7	0.006
SUA, µmol/L	189.6 ± 30.9	253.3 ± 15.8	311.5 ± 16.5	385.1 ± 33.0	0.000
SBP, mm Hg	110.0 ± 11.8	112.8 ± 14.0	117.4 ± 13.6	120.5 ± 12.9	0.003
DBP, mm Hg	72.3 ± 8.0	73.1 ± 8.9	76.2 ± 8.3	77.7 ± 8.3	0.008
PP, mm Hg	70.0 ± 30.6	65.7 ± 32.9	64.5 ± 34.4	67.0 ± 29.4	0.862
TG, mg/dL	127.8 ± 83.8	140.6 ± 75.2	164.7 ± 109.0	179.8 ± 84.3	0.034
TC, mg/dL	126.7 ± 45.5	135.8 ± 41.1	145.6 ± 49.0	155.4 ± 50.3	0.030
HDL-C, mg/dL	44.0 ± 14.0	43.8 ± 15.2	41.5 ± 14.0	38.2 ± 12.4	0.044
LDL-C, mg/dL	67.4 ± 37.8	70.3 ± 40.0	74.8 ± 35.4	87.7 ± 47.5	0.038

Values are presented as mean ± SD. P-values are obtained from one-way ANOVA.

### Prevalence of hypertension and prehypertension in the SUA quartiles

Male and female participants with hypertension and prehypertension in each quartile of SUA are presented in Table 3. The prevalence of hypertension and prehypertension was significantly higher in male (15.4 and 47.6%, respectively) than in the female (5.6 and 33.4%, respectively) subjects (p < 0.01). Both hypertension and prehypertension prevalence was found to be increased in the male-female groups with increasing concentrations of SUA in the quartiles.

**Table 3:** Prevalence of hypertension and prehypertension in the SUA quartiles.

Prevalence	Q1	Q2	Q3	Q4	Total
**Hypertension, %**					
Male	14.9	14.8	15.6	16.3	15.4*
Female	2.9	5.6	6.0	10.0	5.6
**Prehypertension, %**					
Male	47.1	46.8	48.5	48.1	47.6*
Female	22.1	26.4	30.0	55.0	33.4

Blood pressure (mm Hg) was categorized as normal (SBP < 120; DBP < 80), prehypertensive (SBP 120–139; DBP 80–89) and hypertensive (SBP ≥ 140; DBP ≥ 90)^32^. *P < 0.01 when compared to female. P-values are obtained from independent sample t-test.

### Association of SUA with blood pressure

**Figure 1:**
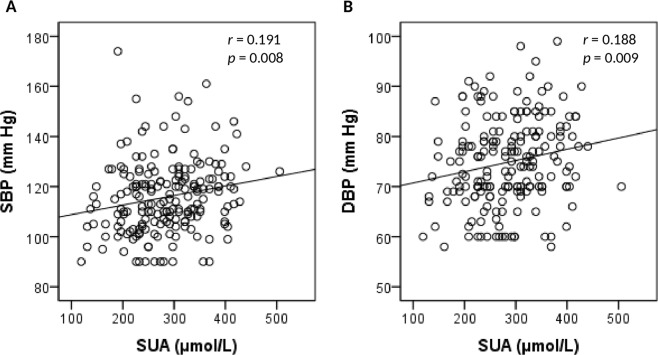
Association of SUA levels with SBP (**A**) and DBP (**B**). The scale in the Y-axis is not similar between the figures.

**Figure 2:**
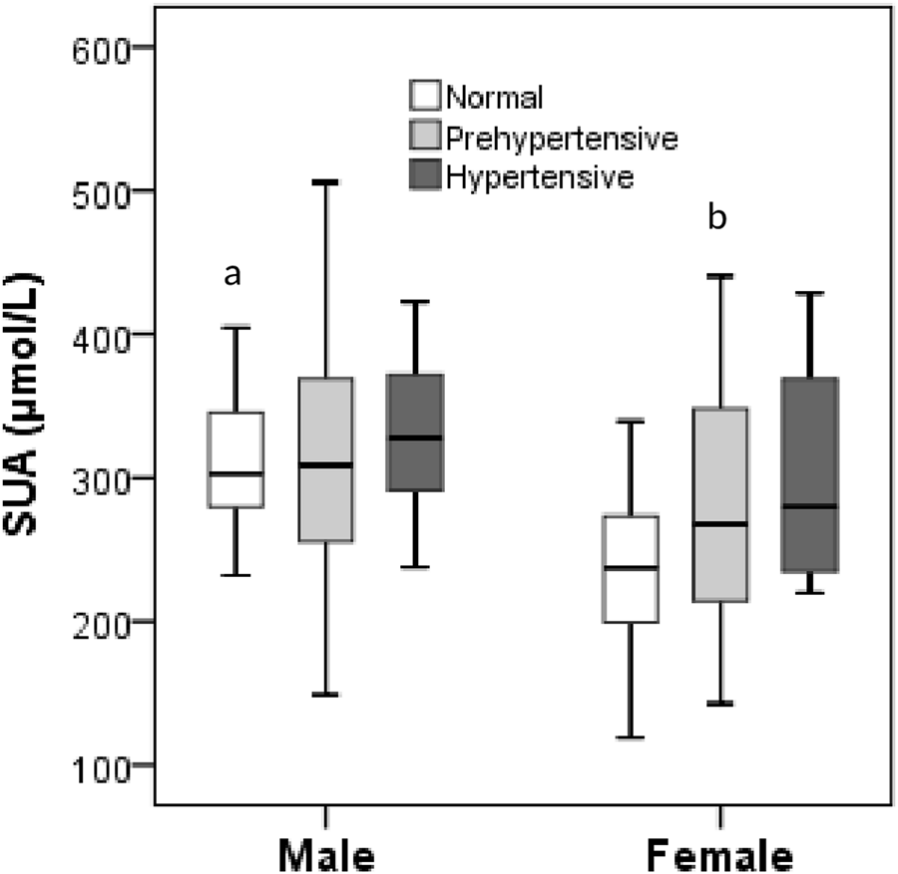
Levels of SUA in the normal, prehypertensive and hypertensive groups by gender. ^a^P < 0.001, when normal blood pressure is compared between male and female group and ^b^P < 0.05 when prehypertension is compared to normal blood pressure in the female group.

SUA concentrations were positively associated (p < 0.01) with both SBP and DBP (Fig. 1). SUA levels were increased positively in hypertension and prehypertension group compared to the normal blood pressure group in both sex groups (Fig. 2). The difference for the SUA levels in the categorized blood pressure groups is more pronounced in the female’s cohort than in the male’s cohort (Fig. 2). In logistic regression analysis, SUA was correlated with hypertension in the SUA quartiles (p < 0.01 for trend) (Table 4). After adjusting for age and sex (model 1), the odds ratios (ORs) (95% CI) were 1.34 (1.10–1.58), 1.98 (1.45–2.40) and 2.80 (1.90–3.70), respectively in Q2 to Q4 compared to Q1. In model 2, after additionally adjusting BMI, the ORs (95% Cl) were 1.25 (1.07–1.44), 1.61 (1.30–1.92) and 2.45 (1.80–3.11), respectively in Q2 to Q4 compared to Q1. In model 3, the correlation remained unchanged even after adjustment of lipid profile.

**Table 4:** Association of SUA quartiles with hypertension.

	OR (95% CI)				p-values for trend
	Q1	Q2	Q3	Q4	
Model 1	1	1.34 (1.10–1.58)	1.92 (1.45–2.40)	2.80 (1.90–3.70)	<0.01
Model 2	1	1.25 (1.07–1.44)	1.61 (1.30–1.92)	2.45 (1.80–3.11)	<0.01
Model 3	1	1.15 (0.94–1.36)	1.37 (1.08–1.66)	1.68 (1.24–2.12)	<0.01

The multiple logistic regressions were done to evaluate the association between SUA quartiles and hypertension. Model 1: age and sex were selected. Model 2: age, sex and BMI were adjusted. Model 3: age, sex, BMI and lipid profile were selected.

## Discussion

The present study reveals a positive association between elevated SUA levels and hypertension in a general adult cohort in Bangladesh. This association was persisted after adjustment for age, sex, BMI, and lipid profile. An increasing trend for the incidence of prehypertension and hypertension was found in both genders with elevated levels of SUA in the quartiles. This is the first study that has evaluated the relationship between SUA and hypertension among Bangladeshi adults.

Some epidemiological studies have demonstrated the relationship between hyperuricemia and hypertension in adult population^24,26,27,35–37^. For example, a study in Japanese adults showed that hypertension OR was 1.20 for each 1 mg/dL increase in SUA concentration after adjusting of multiple confounders, and the ORs in the highest quartile were 1.58 (1.44–1.75) in males and 1.60 (1.39–1.84) in females, compared with the lowest SUA quartile^24^. A study with the cross-sectional design carried out in the US reported that elevated SUA levels were positively associated with prehypertension, and the multivariate OR comparing highest quartile of SUA (>356.9 µmol/L) with the lowest quartile (<237.9 µmol/L) was 1.96 (1.38–2.79)^36^. A study conducted in a large number of hypertension-free individuals in the US reported that the multivariate relative risk was 1.65 (1.41–1.93) when compared the highest quartile of SUA (≥390 µmol/L) with the lowest quartile (≤260 µmol/L)^35^. In Framingham Heart Study, after examining the participants for 4 years, it has been reported that increasing of SUA by each standard deviation was related with an OR of 1.17 (1.02 to 1.33) for developing hypertension and an OR of 1.11 (1.01 to 1.23) for progression in blood pressure^38^. Similar to our findings, in some recent studies, a positive association between SUA and hypertension was found in adult cohorts of China^22,23,39^ and Japan^24,37^.

In this study, we observed comparatively a stronger relationship for SUA concentration with hypertension and prehypertension in female than in the male participants. A systematic review indicates that females with an increased concentration of uric acid are more vulnerable to hypertension than males^40^. Although data suggested that gender difference was correlated with elevated SUA levels in males^41^, further studies investigating the role of sex hormones may help to understand the underline mechanisms of the sex differences. An effective strategy preventing complications of elevated SUA should also focus on sex differences^23^. We also observed that after overall age adjustment, SUA showed significant correlation with SBP and DBP. An association of SUA with blood pressure has been reported in Korean adults aged <60 years and Chinese adults aged 41 to 50 years^42^. The exact mechanism for the age-related relationship between SUA and blood pressure still remains to explore. Whether age related such a relationship is because of the differences in ethnicity, BMI, certain single-nucleotide polymorphism, or oxidative oxygen species needs to be further investigated^22^. The extended mechanism for the effect of SUA on hypertension is yet to be elucidated. There are some hypotheses partly explain the association between SUA and high blood pressure. One of the possible mechanism might be uric acid deposition on the blood vessels walls activates the renin-angiotensin system, suppress the liberate of carbon monoxide, enhance inflammation, and leads to vasoconstriction on later stage, which consequently leads to hyperplasia and incidence of hypertension^2,43–45^. Another possibility involving oxidative stress and endothelial dysfunction associated with high SUA levels may contribute to high blood pressure^46^.

Our study had a few limitations. First, the cross-sectional design of this study may preclude the cause-effect relationships between SUA concentrations and hypertension being assumed. Second, the sample size of this study was relatively small; therefore, the findings may not represent for the whole population of Bangladesh. Third, we did not have individual information on family history of hypertension and physical activity which may affect the incidence of high blood pressure. Moreover, all participants of this study were apparently healthy adults; whether our finding is similar in other ethnic populations needs to be further studied. However, this study findings are worthy as a reference for future investigations. Further studies are required to establish the potential mechanism between SUA and hypertension in humans.

## Conclusions

Increased levels of SUA were positively associated with hypertension among general adults in Bangladesh. The SUA quartiles also showed significant correlation with SBP and DBP. Our study findings suggest an independent relationship of elevated SUA with hypertension and indicate the significance of maintaining normal SUA concentration to prevent hypertension. Early and proper management of SUA levels, as well as blood pressure, may be useful in preventing the development of future CVDs.

## Data Availability

All relevant data are within the paper. The datasets generated and analyzed during the current study are available from the corresponding author upon reasonable request.
